# Creating cognitive normative data for rural Indian population

**DOI:** 10.3389/fnagi.2026.1838843

**Published:** 2026-07-24

**Authors:** Nilima Nilima, Hina Narzari, Shubham Gupta, Kritika Sharma, Maroof Khan, Phaniraj Vastrad, Akshata Nandini, Priya Darshanraj, Sandip Bhattacharjee, Pradyut Datta, Gajraj Singh Shekhawat, Rohan Sharma, Arnav Verma, Chondamma PD, Harshath Ajay V, Jajur Rajappa Sandesh, Ambika Y. V, Shalina Jamatia, Bishakha Sen, Priyanka Saini, Moh Sualeh, Urvashi Sahu, Priyanka Kumari Meena, Hitesh Tiwari, Shaily Bhushan, Sagar PM, Ripanjit Singh, Manish Acharjee, Vikram Raigar, Kalpna Sharma, Aishwarya Bhovi, Mailarappa Padiyappa Hooli, Shilpa K, Naveen MR, Dipankar Deb, Pameli Jamatia, Pooja Sharma, Akhilesh Nagar, Tinku Yogi, Rishav Bhatia, Rishabh Gautam, Sneha Sneha, Kamini Kamini, Sakshi Sharma, Aditi Dubey, Varuna Sharma, Kajal Fulara, Shikha Chaudhary, Kanika Vasudeva, Nimin Hafeez, Chanchal Goyal, Subarna Roy, Manish Barvaliya, Subrata Baidya, Shampa Das, PK Anand, Sunil Kumar Raina, Meenakshi Sharma, R. S. Dhaliwal, Abhik Sinha, Anu Gupta, Venugopalan Y. Vishnu, MV Padma Srivastava

**Affiliations:** 1 All India Institute of Medical Sciences, New Delhi, India; 2 Model Rural Health Research Unit (MRHRU), Raichur, Karnataka, India; 3 Model Rural Health Research Unit (MRHRU), Khumulwng, Tripura, India; 4 Model Rural Health Research Unit (MRHRU), Jaipur, Rajasthan, India; 5 Department of Community Medicine, Dr. RP Government Medical College, Tanda, Himachal Pradesh, India; 6 Indian Council of Medical Research, New Delhi, India; 7 Department of Health Research, Government of India, New Delhi, India; 8 Indian Council of Medical Research- National Institute of Traditional Medicine (ICMR-NITM), Belagavi, Karnataka, India; 9 Division of Non-communicable Diseases, Indian Council of Medical Research, New Delhi, India

**Keywords:** dementia, older-adults, ICMR-NCTB, MCI, normative data, rural, India

## Abstract

**Introduction:**

Population-specific normative data are essential for accurately interpreting cognitive test performance, particularly in low-resource settings characterized by low literacy rates. In India, normative values for the Indian Council of Medical Research-Neurocognitive Tool Box (ICMR-NCTB) among older adults in rural communities are limited. The objectives are to generate education-stratified normative values for ICMR-NCTB cognitive domains and to evaluate their cross-sectional classification ability for identifying early cognitive impairment among high-risk older adults in rural India.

**Methods:**

Baseline data were derived from the Strategic Multimodal Intervention in at-risk elderly Indians for prevention of dementia (SMRUTHI) cohort, a large, multi-site community-based study conducted across four rural regions of India. Neuropsychological assessments were administered to adults aged 55 years or older using the ICMR-NCTB battery. Normative centiles were estimated using quantile regression and the Box-Cox power exponential (BCPE) method to account for skewness and kurtosis in the test score distributions and to adjust for site effects. Receiver operating characteristic (ROC) analyses assessed the ability of individual tests and composite scores to distinguish normal cognition from early cognitive impairment, as defined by the Clinical Dementia Rating-Sum of Boxes (CDR-SOB).

**Results:**

Of 3,917 individuals pre-screened, 3,000 cognitively normal and 175 cognitively impaired participants were included. Education demonstrated moderate associations with performance, whereas the demonstrated of age and gender were minimal. The normative data demonstrate a steady pattern across older age strata, with stable, domain-specific age-related changes rather than an unexpected decline. Literates performed better than their low-literate counterparts. ROC analyses indicated acceptable to good classification performance for several tests, particularly episodic memory measures and the composite z-score (area under the ROC curves up to 0.84). Empirically derived cutoffs approximated the 25th percentile, striking a balance between sensitivity and specificity for screening purposes.

**Discussion:**

The large-scale, community-based study generated, age-education-stratified normative data for the ICMR-NCTB among high-risk older adults in rural India. The findings underscore the importance of population-specific, percentile-based normative values for early detection of cognitive impairment in settings with low literacy and high cultural diversity, while highlighting limitations related to ceiling effects in certain measures.

## 1 Introduction

The number of older adults globally is projected to rise by 22% by 2050 (Naja et al., 2017). Asia’s share of the older adult population is expected to rise from 56.7 to 61.5% between 2020 and 2060 (He et al., 2022), with the proportion of older adults in India expected to double by 2050 (UNFPA in India, 2026). This demographic shift underscores the crucial importance of understanding age-related health issues, particularly those affecting cognitive function. According to the National Institute on Ageing and Alzheimer’s Association (NIA-AA), the syndromal cognitive staging scheme categorizes the cognitive continuum into three categories namely Cognitively Unimpaired (CU), Mild Cognitive Impairment, and Dementia (further divided into mild, moderate, severe) (Jack et al., 2018). CU, often referred to as cognitively normal, implies cognitive performance within expected range for the individual based on available information. MCI implies cognitive performance below expected range for that individual based on all available information. The basis of both CU and MCI could be clinical judgment and/or cognitive test performance, which may involve comparison to normative data, adjustments for age, sex, etc. Dementia involves substantial progressive cognitive impairment that affects several domains and/or neurobehavioral symptoms. The symptoms may be recognized by the individual, the observer, or the observed in longitudinal cognitive testing. Cognitive impairment (MCI or dementia) is known to impact an individual’s health, overall quality of life, and survival, often leading to higher rates of disability among older adults (Gill et al., 2020). Nationally representative studies claim approximately 8.8 million (7.4%) older Indian adults live with dementia, with cases unevenly distributed across states and place of residence (Lee et al., 2023).

Despite this significant burden, only a limited number of empirical, population-based studies have investigated cognitive function and its multifaceted associations with demographic, socioeconomic, health, and behavioral factors, especially in low-resource setting (Muhammad et al., 2022; Prince et al., 2012). One important barrier is the availability of culturally appropriate and linguistically adapted neuropsychological tests with robust normative data (Ardila, 2005). Normative data are observational, empirical, statistical data summarizing what is usual (or typical) in a defined population, culture, institution, or health care system at a specific point or period of time, and describe observed phenomena or characteristics (Schmidt and Pardo, 2014). Selecting appropriate normative data affects the accuracy of neuropsychological assessment findings, which may further reduce false diagnoses of cognitive decline (delCacho-Tena et al., 2024). The Indian population is heterogeneous not only in terms of language and religion but also in a myriad of cultural and socioeconomic factors that may confound performance on neuropsychological tests (Ganguli et al., 1996; Menon et al., 2020; Verma et al., 2021). Cognitive testing is particularly challenging in rural areas where formal education, healthcare access, and validated normative benchmarks are lacking, leading to delayed diagnosis and an increased risk of misclassification when normative values validated for urban or literate populations are applied (Ganguli et al., 1996; Verma et al., 2021). The literature reports that older adults living in rural areas have significantly lower cognitive performance than those living in urban communities (Muhammad et al., 2022; Muhammad, 2023; Saenz et al., 2018). The considerable sociocultural and demographic heterogeneity of the Indian population highlights the need for culturally appropriate cognitive assessment tools and language-specific normative data, particularly for those who remain under-represented in neuropsychological research.

Cognitive assessment tools available in Indian context include NIMHANS Neuropsychological Battery for Elderly (NNB-E), Addenbrooke’s Cognitive Examination-III (ACE III) (Bhattacharyya et al., 2025; Mekala et al., 2020), the Consortium to Establish a Registry for Alzheimer’s Disease-Neuropsychological Battery (CERAD-NB) (Welsh et al., 1994), the Kolkata screening battery (KS) (Das et al., 2006), and the PGI battery of brain dysfunction (PGI-BBD) (Pershad and Verma, 1990), Computerized assessment of adult information processing (COGNITO), and the Indian Council of Medical Research-Neurocognitive Tool Box (ICMR-NCTB) (Iyer et al., 2020; Verma et al., 2021) battery. ICMR-NCTB was developed upon reviewing existing Indian and international batteries (including ACE-III, CERAD, NIMHANS, Kolkata Screening Battery, PGI-BBD, etc.) and selecting culturally adaptable tests across Indian languages. NNB-E is considered the closest to ICMR-NCTB, however, NCTB adds multilingual standardization and harmonized multi-domain assessment protocol across India validated for education levels and multiple languages including Hindi, Bengali, and Kannada making it more generalizable. Thus, determining expected cognitive functioning using the ICMR-NCTB battery and establishing population-specific normative data (for comparison with individuals suspected of dementia) is the need of the hour for the rural older-adults (Freedman and Manly, 2015; Mitrushina et al., 2005).

Previous studies employed validation approaches estimating diagnostic accuracy, sensitivity, specificity, and reported optimal cut-offs for dementia or MCI classification, including mostly urban-residing literate participants from a hospital-based setting (Menon et al., 2020; Verma et al., 2021) Although one of the studies (Verma et al., 2021) demonstrated good diagnostic accuracy for dementia across multiple Indian languages, it did not provide centile-based normative values, with an underrepresentation for low-literacy rural participants (71% urban). Despite establishing diagnostic accuracy and cross-cultural applicability, several important limitations remain unresolved, such as the unaddressed skewed and kurtotic distribution commonly observed in neuropsychological test scores. Given the paucity of studies reporting population-specific norms for the ICMR-NCTB tests in India, this study aims to fill a substantial gap by generating norms for the NCTB, particularly adapted for people residing in rural communities who are not formally educated. The objective of this study was to generate normative values and assess the ability of the ICMR-NCTB tests to classify individuals with normal cognition from those with early cognitive impairment.

Hospital-based studies validating the ICMR-NCTB test battery and assessing the classification of the older-adults with MCI suggested that language may influence test performance distributions, with non-literate participants performing poorly (Menon et al., 2020; Sosa et al., 2009). Literature suggests age, gender and literacy as the most robust and consistently reported determinants of neuropsychological test performance in India (Ganguli et al., 1996; Kahali et al., 2023; Waldrop-Valverde et al., 2015). Although occupation, socioeconomic status and nutrition may influence cognition, education is considered a surrogate (Ganguli et al., 1996). Age and literacy, as primary stratification variables, therefore represent a balance between epidemiological relevance, statistical robustness and practical applicability.

Establishing reliable and valid normative data requires careful methodological consideration, often favoring hybrid regression and stratification models to reduce bias (O’Connell et al., 2022). As the majority of ICMR-NCTB test scores commonly exhibit skewness and kurtosis (delCacho-Tena et al., 2024; Sachs et al., 2020), conventional methods for centile estimation may not provide an accurate and precise estimate (delCacho-Tena et al., 2024; O’Connell et al., 2022; Sachs et al., 2020). A quantile regression (O’Connell et al., 2022) and the Box-Cox power-exponential (BCPE) method account for the skewness and kurtosis inherent in the data.

To the best of our knowledge, this is among the first nationally representative, large-scale, community-based efforts to present education- age-, language- age-, and language-education-specific normative values across diverse cognitive domains among high-risk older-adults enrolled in a large-scale community-based cohort in rural India. The main aim of this study was to generate regression-based normative values for cognitive tests in a large community-based sample of high-risk older adults residing in rural India. By generating the indicators of normative cognitive decline that are relevant to low-resource settings, this study aligns with the sustainable development goals of *good health and well-being* (countries Un-Riwaa, 2025) and *reduced inequalities* (The United Nations Department of Economic and Social Affairs [UN DESA], 2025), while also supporting the World Health Organization (WHO) Decade of Healthy aging initiative (WHO, 2026).

## 2 Materials and methods

Baseline data from SMRUTHI, a multicentre, large, representative cohort study designed to assess dementia risk among older adults enrolled from four sites in India was collected through face-to-face in-home interviews. The study was conducted across four sites representing the North (Himachal Pradesh), South (Karnataka), East (Tripura), and West (Rajasthan) zones of India. For the development of regionally relevant normative values, the geographically and culturally diverse states were included to enhance the generalizability, ensuring a culturally (linguistically) diverse population with different aging patterns and literacy. At each study site, cognitive assessments were administered in the predominantly spoken language to ensure that participants could communicate comfortably. Individuals were classified to be at high-risk of dementia if their Cardiovascular Risk Factors, Aging, and Incidence of Dementia (CAIDE) score was ≥ 6, a threshold that indicated a moderate to high presence of modifiable vascular and lifestyle risk factors and entails **≥** 2% risk of developing dementia within 20 years (Kivipelto et al., 2006). The cutoff (CAIDE score ≥ 6) was also used by some landmark clinical trials of multidomain lifestyle intervention for dementia prevention like the FINGER study (Kivipelto et al., 2013). Details of the SMRUTHI data collection can be found in the protocol published elsewhere (Gupta et al., 2025).

### 2.1 Measures

While designing SMRUTHI, the cognitive assessments were selected for their psychometric properties after a thorough literature review, their availability in multiple Indian languages, their relevance to those aged 55 and above, and their time and cost of administration, among other factors. The ICMR-NCTB battery provides scores for cognitive domains such as episodic memory, executive function, language, and visuospatial function. The ICMR-NCTB is available in versions adapted for literate and illiterate individuals. In this study, not literate and low literate were defined as the individuals with less than eight years of formal school education and literates were the ones with eight or more years of formal school education. The tests employed in the present study were the Verbal Learning Test (VLT), Picture Naming Test (PNT), Trail Making Test (TMT), Category Fluency Test (CFT), Test desn Neuf Images du 93 (TNI-93), and Stick Design Test (SDT). While VLT, CFT, TNI-93 and SDT are similar in literate and illiterate versions, there are adaptations to TMT (pointing to fingers instead of pen and pencil task) and PNT (colored pictures instead of black-and-white line drawings) in the illiterate version (Iyer et al., 2020). Administration of this battery usually takes approximately 45 minutes (min) to an hour. The tests were administered at sites in the participants’ respective languages; however, for norm generation, the data were treated as homogeneous.

TMT is a measure of attention, speed, and mental flexibility. It also tests spatial organization, visual pursuit, recall, and recognition. This test is time bound where participants have to complete the test as quickly as possible. It has two parts TMT A and TMT B which are time bound and the time needed to complete test in seconds is marked as the score. In the illiterate version, TMT A requires the individual to draw their fingers from one circle to the next in ascending order, with ten numbers distributed across the page. The first and last numbers are marked with green and red dots to indicate the start and end of the test. In the literate version of TMT A, the participant is required to join the numbers in ascending order from 1 to 25 with a pen or pencil. This test measures visuomotor speed, numeric sequencing, and visual scanning. In TMT B, the procedure is similar, except that the individual has to connect the numbers alternately between two colors (black and white). TMT B measures the same skill sets as TMT A, along with mental flexibility. Thus, TMT B usually takes longer to complete due to its complexity. While administering TMT, the time taken (in seconds) to complete the test is scored accordingly. The TMT A and TMT B pictures are colored for the illiterate population, and black and white for the literate population. VLT measures episodic memory, assessing immediate and delayed recall and recognition memory. In this test, ten unrelated words are presented verbally to the participants across three learning trials (T1, T2, T3), followed by a delayed recall assessment after 20 min. Trials T1, T2, T3 are scored from 0 to 10 and total learning score is the sum of all three trials (ranging from 0 to 30). Delayed recall is scored from 0 to 10. For delayed recognition assessment, 10 previously learnt words along with 10 new words are verbally presented to the participants for recognizing the 10 previously learnt words (score ranges between 0 to 20 based on correct identification). The test lasts 20–30 min. This test has been translated into Hindi, Bengali, and Kannada by previous researchers (Iyer et al., 2020; Verma et al., 2021), and the translated versions are being used in current research. PNT is a test which assesses word-finding ability, language, and semantic memory. The participants have to produce names for 30 drawings presented. These drawings are colored for the illiterate population and in black and white for the literate population. Each picture is shown in the predetermined order, one at a time, and the participant is given 10 s to name the picture (if correctly named a score 3 is assigned). If the individual is unable to identify the picture within 10 s, a category cue is given (if identifies correctly after category cue, a score 2 is assigned). If, within 10 s of providing the cue, the subject makes an error, a phonemic cue is provided (if identifies correctly after phonemic cue, a score 1 is assigned). If the subject makes an error after a maximum of 10 s post the phonemic cue, a score zero is assigned and the psychologist moves on to the next picture. The maximum time allowed for each picture is 30 s. The duration of this test is from 3 to 10 min. Total PNT score is a sum of scores assigned for each picture, ranging between 0 and 90. CFT measures executive function, semantic memory, and language. In this test, a semantic category is given to the participant, and they are asked to generate words within 1 min in each category. It includes three categories: animals, vegetables, and food. The total score is the total number of correct words generated, with no specified upper threshold. TNI-93 is a test of episodic memory which has two stages: encoding and recall. During the encoding stage, nine pictures are shown to the participants, and they are asked to remember their names and locations. To assess immediate recall, participants are asked to recall the names of the pictures. If a participant is unable to recall any picture, semantic cues are provided. The pictures are shown again if the participant fails to recall, even with the semantic cues. Following this, the participant is asked to count backwards by three from 40 for five trials as an interpolated/distractor task. Then, recall stage is followed where participants have to recall the names of as many pictures as possible within 2 min (free recall). For any missed items, semantic cues are given (cued recall). The total recall is the sum of free and cued recall (ranges from 0 to 9). To assess spatial recall, nine pictures are shown to the participant one by one, and they have to locate them on a blank grid. The test usually lasts for about 10 min. The spatial recall score is the number of correctly located pictures on the grid (ranges between 0 and 9). SDT is a test which examines visuo-constructional ability by assessing visuo-spatial memory and visuo-spatial neglect (Baiyewu et al., 2005). In this test, the participant must use wooden sticks to copy a picture created by the examiner. The participant has to recall and construct the figure again twice after 3 and 20 min. The duration of the test is usually 30 min. Each trial is scored from 0 to 10 and total SDT score is the sum of three trials (ranging from 0 to 30). Besides this, completion time in seconds is also noted in each trial.

Since the TMT-A and TMT-B are timed tests, a lower score indicates better executive function. To maintain consistency with the other cognitive measures, we reverse-coded and normalized the Trail A and B score for further analysis (Perry et al., 2022; Rosenberg et al., 2018). A z-score (z = (y - mean)/standard deviation) was calculated for each test measure (Y), and the scores were then averaged to obtain domain-specific and overall scores, such that higher scores indicate better cognitive performance.

### 2.2 Participants and study flow

The data were collected between April 2024 to December 2025. Upon explaining the purpose of this study, household volunteers were pre-screened to determine initial eligibility. If eligible in pre-screening (age ≥ 55; absence of significant hearing or vision impairment; and permanent residency within the household), an informed consent was obtained and the first visit was scheduled where screening form was duly filled to assess the detailed eligibility (CAIDE score ≥ 6; and not having any pre specified ailments (Gupta et al., 2025)). An individual who consented to be a part of the study, completes screening and meets detailed eligibility criteria was considered enrolled.

Of the 3,917 individuals pre-screened, 3,550 consented to undergo neuropsychological assessments and agreed to participate for the entire study duration (study participation rate of 90.6%). To establish the cohort of at-risk older-adults, 177 were excluded for a CAIDE score < 6. 124 (3.5% of those consented) participants were ineligible due to having one or more of the following known conditions: any stroke (35), intellectual disability (05), dementia (11), epilepsy (02), psychiatric illness such as major depressive disorder (03), structural brain abnormality (02), psychosis (04), systemic malignancy (5), end-stage renal disease (02), decompensated liver disease (01), and significant vision, hearing, communication, and head injury impairments affecting neuropsychological tests (29, 17, 32, and 07, respectively). Finally, 3,249 eligible and willing participants were included in the cohort; however, 3,000 were assessed as cognitively normal (CDR sum of boxes score of zero), 175 were cognitively impaired (CDR sum of boxes score more than zero) and 74 had missing CDR-SOB values.

### 2.3 Statistical analysis

The quality check included, but was not limited to, checking the consistency of the data; missing values where they were not possible (viz., gender, age); characters entered where a numeric value was expected; and outlying or extreme values due to data entry mistakes. Data consistency was investigated for variables where the same value was expected. Outlying and extreme values of numeric variables were discussed with the experts to check whether they were within their plausible ranges. They were then sent to the respective sites for further checking of the hard copies. To ensure standardized cognitive test scores, psychologists on the central team reviewed the scanned documents to assess the correctness of the neuropsychological testing conducted at sites.

Data were summarized as mean ± SD, median (interquartile range), and frequency (%) as applicable. After assessing the distributional assumption, a *t*-test or Mann-Whitney U test was used for intergroup comparisons (men vs women; illiterate and low literate vs. literate) presented in Supplementary Table S1. The chi-square test was used to investigate associations. Point-biserial and Spearman’s correlation coefficients were used to investigate relationships between test scores and education, gender, and age, respectively. A correlation coefficient of |0.15| or greater was considered significant in this study. Skewness and kurtosis values, along with a histogram grid plot, are presented for each test score. Several cognitive measures are prone to error due to participants’ inability to read, write, or understand the instructions; education serves as a proxy for this inability. Given systematic differences in measurement performance associated with higher age, being women, mostly indoor activity, limited exposure to literary activities, lower or no education, and language barriers, a regression model adjusting for age, gender, site, and education was applied to the full dataset. It is important to note that a different version of the ICMR-NCTB battery was used among *the literate*, *low-literate, and non-literate groups*, resulting in non-equivalent measurement and necessitating adjustment for education to reduce version-related bias. The findings of regression model adjusting for age, gender, site and education were comparable across education levels, age, gender, and culture/language. Given that hybrid regression and stratification models perform best at reducing bias (O’Connell et al., 2022), another set of regression models were run, adjusting for the effect of gender and site on each cognitive test within the pre-specified strata of education (*literate*, *low literate, and not literates*) and age in years (55–64; 65–74; 75 and more), and a minimum of 100 individuals (Mannar et al., 2024) was maintained in most scenarios.

### 2.4 Quantile regression model estimation for individual cognitive tests

Given the skewed and kurtotic nature of the cognitive test scores, quantile regression (commonly known as median regression) was used instead of multiple linear regression. Quantile regression fits separate models for specified quantiles (5th–95th percentiles), such as the 50th percentile of TMTA at ages 55–59 years, which was directly obtained from the 0.5 quantile model. Quantile regression is not a likelihood-based model; therefore, likelihood ratio tests or Akaike Information Criterion/Bayesian Information Criterion (AIC/BIC) for model comparisons were not applicable.

### 2.5 Model comparisons using the 95% inter-percentile range

Six models were considered for comparison of the best fit. The models included the main effects of age, gender, and education; the age-gender interaction; the age-education interaction; the education main effect with the age-gender interaction; the gender main effect with the age-education interaction; and the gender-education interaction. Two additional models, with age and gender as main effects, one among literates and another among low-literate and non-literate groups, were compared to assess whether education-specific data offer any additional advantage over the full data analysis. A model with the most precise estimates of the 5th percentiles was chosen to obtain the standardized residuals and transform them into normative data. To obtain the 95% interpercentile range, 1,000 bootstrap resamples were run on the predicted scores. For each bootstrap sample, the 5th percentile of the predicted scores was obtained (i.e., the fitted/predicted 5th percentile value). A sampling distribution of these 1,000 5th percentiles yields the 2.5th and 97.5th percentiles, resulting in a 95% Inter-Percentile Range (IPR) (width of the interval 2.5th, 97.5th centile) for the test score. A *p* < 0.05 was considered significant throughout.

### 2.6 Quantile regression with sites as a fixed effect

It is noted that the four sites in SMRUTHI-INDIA were not a random sample. They were functional groups that facilitated data collection from diverse locations across India and aided generalizability. However, owing to the observed between-site variability, the assessments were made to utilize appropriate adjustments. Quantile regressions were estimated at the 5th, 10th, 15th, 25th, 50th, 75th, 90th, and 95th percentiles, potentially yielding quantile-specific between-cluster effects. Additionally, cluster-robust inference is an asymptotic method that can yield severely biased downward standard errors in cases with a small number of clusters. To account for unobserved heterogeneity (possibly due to administrative, assessor, recruitment, or cultural differences) and to avoid the large-k asymptotic bias, the site indicators were treated as fixed effects in the appropriate quantile regression model to estimate within-cluster effects.

### 2.7 Box-Cox power-exponential method

Quantile regression is a distribution-free method, whereas the BCPE approach assumes that the outcome follows a parametric distribution after transformation. BCPE explicitly models skewness and kurtosis, building on the Box-Cox transformation, and converts skewed and kurtotic measures into a Box-Cox Normal distribution, which is then used to run parametric models to obtain normative values.

BCPE centiles were used as a reference for evaluating growth among older adults. The challenge in constructing centile curves is ensuring the smoothness of percentiles over age. Smoothing is necessary to retain short-term fluctuations in cognitive abilities among older adults. The fundamental aspect of centile estimation is the z-score, and the interpretation of z-scores is straightforward in terms of percentiles when the y-values follow an exact normal distribution, i.e., the frequency curve of scores should be unimodal, symmetrical (skewness = 0), and with a normal peak (kurtosis = 3). However, the majority of the ICMR-NCTB tests for each age were skewed and kurtotic, making it suitable to employ a BPCE method, which has four parameters: median, coefficient of variation, box-cox transformation parameter lambda, and a kurtosis parameter Tau. BPCE, also known as the LSM-P, is a generalization of the well-known LSM method (Cole and Green, 1992; Indrayan, 2014). Preliminary model diagnostics indicated that modeling scale (σ), skewness (ν), and kurtosis (τ) as a function of age did not substantially improve fit for most outcomes (the generalized Akaike information criterion (GAIC) change was within two units); therefore, only μ was modeled as a smooth function of age, simplifying the model while preventing over-fitting. All models were adjusted for study site as a fixed effect in the location parameter. Site-adjusted centiles were derived by averaging predictions across sites, weighted by their observed sample proportions. Generalized Additive Models for Location, Scale, and Shape (GAMLSS) is a function in the *gamlss* package for R (version 4.3.1) that provides a modeling framework for the BCPE. It was used to generate the centile plot and the predicted centile values.

## 3 Results

The participant characteristics by gender and education are presented in Supplementary Table S1. A statistically significant difference in baseline characteristics was noted; however, the differences were considered clinically insignificant.

The data were assessed for normality using the Shapiro-Wilk test, and the absolute measures of skewness and Kurtosis were calculated. Histograms were generated to assess the type of skewness, and they revealed that the ICMR-NCTB test scores were left-skewed across most ages. The study participants underwent the ICMR-NCTB tests using *low literate or illiterate* [2,563(85.4%)] and the *literate* [437(14.6%)] versions based on their education status. No correlation was observed between age and the test scores; however, education is mild to moderately associated with the test scores, except trail making B, cf vegetables, and cf food items. Education was considered a stratifying variable owing to its high skewness (85% of study participants were *low-literate or nonliterate*) and its correlation with test scores. Gender was not associated with test scores, except for CF animals and PNT.

**FIGURE 1:**
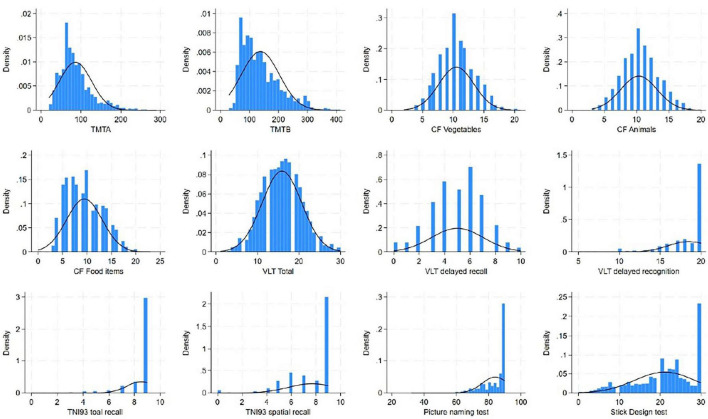
Grid plot of histograms depicting the distribution of the data on ICMR-NCTB tests among older adults in rural India.

Figure 1 and Table 1 highlight the skewed and kurtotic nature of most cognitive test scores and present the bivariate associations between demographic variables and test scores. Education was mildly to moderately correlated with the test scores. However, gender showed a mild correlation, and age showed almost no relationship with performance on the test scores. CF tests were normally distributed, with skewness near zero and kurtosis 3.0. However, CF food items raised concerns about being moderately right-skewed and having lighter tails than a normal distribution, as depicted in Figure 1, leading to the choice of quantile regression and BCPE centiles for all test scores under investigation. Raw percentiles are presented in Table 2.

**TABLE 1:** Skewness, kurtosis, and point biserial correlation of test scores with age, gender, and education among older adults in rural India.

ICMR-NCTB	Skewness	Kurtosis	Gender (r_*pb*_)	Education (r_*pb*_)	Age (r)
TMTA (*n* = 2,554)	1.39	5.60	0.003	−0.117	0.031
TMTB (*n* = 1,643)	1.06	3.81	0.088	0.024	0.011
CF animal (*n* = 2,879)	**0.22**	**2.95**	**0.152**	0.100	−0.016
CF food (*n* = 2,879)	0.45	2.54	0.030	−0.039	−0.031
CF vegetable (*n* = 2,878)	**0.27**	**3.05**	−0.030	0.042	−0.082
VLT total (*n* = 2,880)	0.54	3.61	0.091	**0.334**	−0.040
VLT del recall (*n* = 2,879)	0.18	2.77	0.068	**0.282**	−0.035
VLT del recog (*n* = 2,880)	−1.97	6.80	0.129	**0.198**	−0.046
TNI 93 total (*n* = 2,872)	−3.13	13.78	0.083	0.119	−0.073
TNI 93 spatial (*n* = 2,870)	−1.68	5.98	0.090	0.109	−0.063
PNT (*n* = 2,875)	−1.65	6.19	**0.224**	**0.244**	−0.060
Stick design (*n* = 2,221)	−0.61	2.55	0.135	**0.205**	−0.051

Bold values indicate variables suggesting approximate symmetry, and correlation coefficients indicating at-least mild linear relationship.

**TABLE 2:** The raw normative values of ICMR-NCTB test scores among older adults in rural India.

ICMR-NCTB test scores	Percentiles							
	5th	10th	15th	25th	50th	75th	90th	95th
TMTA (*n* = 2,667)	170	136	121	101	78	62	43	38
TMTB (*n* = 1,703)	289	253	227	195	134	99	83	75
CF vegetable (*n* = 2,998)	6	7	8	9	10	12	14	15
CF animal (*n* = 2,998)	6	7	7	8	10	12	14	15
CF food (*n* = 2,998)	4	5	5	6	9	12	14	16
VLT total (*n* = 2,999)	8	10	11	12	16	19	22	24
VLT D recall (*n* = 2,998)	2	2	3	4	5	6	7	8
VLT D recognition (*n* = 2,999)	13	15	16	18	20	20	20	20
TNI93 total (*n* = 2,989)	6	7	8	9	9	9	9	9
TNI93 spatial (*n* = 2,987)	4	5	6	6	9	9	9	9
PNT (*n* = 2,993)	68	72	75	80	88	90	90	90
Stick design (*n* = 2,294)	7	10	12	17	22	28	30	30

### 3.1 Site as fixed-effects

The present study utilized four sites for data collection; however, to account for potential differences across sites, arising from administration procedures, assessor effects, recruitment strategies, dietary factors, or other unobserved site-specific influences, comparative model assessments were conducted. Two linear regression models were utilized: one without a site component and one with site as a fixed effect. Model fit indices indicated a substantially better fit for the model including site as a fixed effect (AIC = 26,611; BIC = 26,653; *R*^2^ = 20%) versus the model without a site component (AIC = 27,148; BIC = 27,172; *R*^2^ = 1.5%). The model with site as a fixed effect had the lowest AIC and BIC and the highest coefficient of determination, indicating that site accounted for a substantial amount of variance in test performance. In the model without the site component, education had a large effect on performance, sex had a moderate effect, and age was not statistically significant, with limited overall explanatory power. In the model with site effects as fixed, gender and education effects eventually disappeared, and age was statistically significant. The pattern suggested that a substantial portion of the variance previously attributed to demographic variables was absorbed by site-level differences. Thus, to account for systematic differences related to administration, assessor characteristics, recruitment practices, and broader contextual or cultural factors associated with each site, a model with site as a fixed effect was considered.

### 3.2 Model comparisons using the 95% inter-percentile range

A 95% IPR was obtained for each model, run separately for the 12 cognitive impairment tests, as presented in Table 3. The width of these IPRs was then compared across the first six models (M1–M6) to select the model with the best fit, and the related findings were utilized in reporting the normative values, presented age-wise in Table 4 and education-wise in Table 5. Further, the education-specific model was compared with the first six models. It was noted that among literates, none were among the best fit (none had the narrowest 95% IPR). However, among *low-literate or non-literate individuals*, the education-specific model was a good fit for VLT delayed recall after model M6. Thus, education and age-specific normative values are also reported in Table 6.

**TABLE 3:** The 95% Inter percentile range (2.5th, 97.5th centile) for the 5th percentile estimate obtained using quantile regression.

Predictors in the regression model	M1: Age, Gender, Education	M2: Age, Gender	M3: Age, Education	M4: Age, Education, Gender	M5: Age, Gender, Education	M6: Measures correlated with the test score Gender, Education	Education specific-literates	education specific-low literate and not literates
Intersection ICMR-NCTB tests	None	Age Gender	Age Education	Age Gender	Age Education	Gender Education	–	−
TMTA (*n* = 2,667)	4.50 28.50, 33.00	5.16 32.27, 37.43	8.53 24.47, 33.00	5.93 27.07, 33.00	8.57 24.43, 33.00	**4.00** **29.00, 33.00**	7.67 16.64, 24.31	5.13 31.87, 37.00
TMTB (*n* = 1,703)	9.25 52.18, 61.43	14.4 58.17, 72.57	9.02 51.33, 60.35	9.60 51.80, 61.40	8.10 51.90, 60.00	**7** **52, 59**	27.08 26.92, 54.00	11.03 67.97, 79.00
CF vegetable (*n* = 2,998)	1.35 4.65, 6.00	1.50 4.50, 6.00	1.35 4.65, 6.00	1.50 4.50, 6.00	1.37 4.63, 6.00	**1.00** **5.00, 6.00**	1.36 4.64, 6.00	2.00 4.00, 6.00
CF animal (*n* = 2,998)	0.91 4.09, 5.00	0.92 4.08, 5	0.79 4.21, 5.00	0.92 4.08, 5.00	0.91 4.09, 5.00	**0** **5.00, 5.00**	2.30 3.70, 6	0.67* **4.65, 5.32**
CF food (*n* = 2,998)	**0** **4.00, 4.00**	0.10 3.90, 4.00	0.03 3.97, 4.00	0.30 3.70, 4.00	0.12 3.88, 4.00	0 4.00, 4.00	1.05 3.00, 4.05	0 4.00, 4.00
VLT total (*n* = 2,999)	1.64 4.83, 6.47	1.74 5.00, 6.74	2.00 5.00, 7.00	1.71 4.82, 6.53	1.68 4.88, 6.56	**1** **6.00, 7.00**	5.26 6.74, 12.00	1.90 6.10, 8.00
VLT D recall (*n* = 2,998)	1.06 −0.06, 1	0.92 0.00, 0.92	1 0.00, 1	**0.72** **−0.06, 0.66**	1.04 **-**0.04, 1	1 0.00, 1.00	2.00 0.00, 2.00	1.67 0.33, 2.00
VLT D recog (*n* = 2,999)	1.48 **9.77, 11.25**	1.58 9.73, 11.31	2.28 9.72, 12.00	1.50 9.83, 11.33	1.54 9.70, 11.24	2 10.00, 12.00	4.00 12.00, 16.00	2.12 10.21, 12.33
TNI93 total (*n* = 2,989)	**1.02** **3.13, 4.15**	1.23 3.00, 4.23	1.03 3.30, 4.33	1.17 3.00, 4.17	1.08 3.00, 4.08	1 4.00,5.00	3.00 5.00, 8.00	1.37 3.87, 5.24
TNI93 spatial (*n* = 2,987)	1.68 0.55, 2.23	1.90 0.32, 2.22	1.81 0.59, 2.40	1.84 0.31, 2.15	1.90 0.45, 2.35	**1** **2.00, 3.00**	2.00 3.00, 5.00	2.17 1.83, 4.00
PNT (*n* = 2,993)	4.18 56.67, 60.85	5.63 55.00, 60.63	4.16 56.24, 60.40	5.49 55.18, 60.67	5.11 55.62, 60.73	**4.00** **60.00, 64.00**	9.32 72.00, 81.68	4.90* 60.77, 65.67
Stick design (*n* = 2,294)	2.13 3.87, 6.00	1.92 4.08, 6.00	2.00 4.00, 6.00	3.22 3.78, 6.00	2.00 4.00, 6.00	**1.00** **5.00, 6.00**	11.16 4.84, 16.00	2.35 4.65, 7.00

*Compared to M1 (the best fit is M6 followed by M3 which had education interaction in the model thus next best model was chosen). Each cell consists of the 95% IPR width and the 2.5th, 97.5th centile values used to obtain the width. Bold values indicate the 95% IPR width of the chosen model.

**TABLE 4:** Age-wise normative values of ICMR-NCTB test scores obtained from appropriate quantile regression model among high-risk older-adults in rural India.

ICMR-NCTB tests	Age (years)	Mean NCTB test score ± standard deviation	Percentiles							
			5th	10th	15th	25th	50th	75th	90th	95th
Trail making test part A (*n* = 2,667)	55–59	75.5 ± 16.0	132	123	112	97	75	62	50	43
	60–64	77.8 ± 15.1	148	123	112	100	78	64	60	48
	65–69	77.3 ± 15.1	132	123	112	100	76	64	60	48
	70–74	77.6 ± 14.3	132	123	112	100	76	64	60	48
	75–79	76.6 ± 17.1	135	123	112	100	76	62	50	43
	80 +	76.0 ± 16.7	132	123	112	100	76	64	60	50
Trail making test part B (*n* = 1,703)	55–59	140.1 ± 31.5	249	227	214	201	154	117	86	77
	60–64	145.4 ± 30.1	253	229	215	201	154	117	88	80
	65–69	144.9 ± 30.9	255	233	215	201	154	117	91	82
	70–74	145.6 ± 29.5	259	239	227	207	153	116	94	85
	75–79	143.9 ± 33.1	260	240	228	208	153	114	96	87
	80 +	143.2 ± 36.3	261	235	218	201	153	113	100	90
CF vegetables (*n* = 2,998)	55–59	10.5 ± 1.3	7	8	9	9	10	11	14	15
	60–64	10.6 ± 1.3	7	7	8	9	10	11	14	15
	65–69	10.5 ± 1.3	6	6	7	8	10	11	14	15
	70–74	10.6 ± 1.3	7	8	9	9	10	12	14	15
	75–79	10.5 ± 1.2	7	8	9	9	10	11	14	15
	80 +	10.2 ± 1.1	6	6	7	8	10	11	13	14
CF animals (*n* = 2,998)	55–59	10.3 ± 1.8	7	8	9	9	10	12	13	14
	60–64	10.4 ± 1.8	7	8	8	9	10	12	13	14
	65–69	10.4 ± 1.8	6	7	8	9	10	12	13	14
	70–74	10.5 ± 1.8	7	8	9	9	10	12	13	14
	75–79	10.5 ± 1.7	7	8	9	9	10	12	13	14
	80 +	10.1 ± 1.6	6	7	8	9	10	11	13	14
CF food items (*n* = 2,998)	55–59	9.2 ± 2.9	5	6	6	7	9	10	11	12
	60–64	9.4 ± 2.9	5	6	6	7	9	10	12	14
	65–69	9.2 ± 2.9	5	6	6	7	9	10	11	12
	70–74	9.5 ± 3.0	5	6	6	7	9	10	12	14
	75–79	9.3 ± 2.7	5	6	7	7	9	10	12	14
	80 +	8.8 ± 2.7	5	6	6	7	8	9	11	12
VLT total learning (TL) (T1 + T2 + T3) (*n* = 2,999)	55–59	15.8 ± 2.5	10	11	12	13	16	19	21	23
	60–64	15.5 ± 2.3	10	11	12	13	16	19	21	23
	65–69	15.7 ± 2.3	10	11	12	13	16	19	21	23
	70–74	15.4 ± 2.3	9	11	12	13	16	19	21	23
	75–79	15.6 ± 2.3	7	9	10	13	16	19	21	23
	80 +	15.8 ± 2.3	10	11	12	13	16	19	21	23
VLT delayed recall (*n* = 2,998)	55–59	4.9 ± 0.9	3	3	4	4	5	6	7	8
	60–64	4.8 ± 0.9	1	3	3	4	5	6	7	8
	65–69	4.8 ± 0.9	3	3	4	4	5	6	7	8
	70–74	4.8 ± 0.9	1	3	3	4	5	6	7	8
	75–79	4.8 ± 0.9	1	3	3	4	5	6	7	8
	80 +	4.9 ± 0.9	3	3	4	4	5	6	7	8
CF food items (*n* = 2,998)	55–59	9.2 ± 2.9	5	6	6	7	9	10	11	12
VLT delayed recognition (*n* = 2,999)	55–59	19.2 ± 0.9	16	17	17	17	19	20	20	20
	60–64	19.2 ± 0.9	15	17	19	20	20	20	20	20
	65–69	19.2 ± 0.9	16	17	19	20	20	20	20	20
	70–74	19.1 ± 0.9	15	17	17	17	19	20	20	20
	75–79	19.2 ± 0.9	15	17	17	17	19	20	20	20
	80 +	19.3 ± 0.8	16	17	19	20	20	20	20	20
Tni 93 total recall (*n* = 2,989)	55–59	8.9 ± 0.08	9	9	9	9	9	9	9	9
	60–64	8.7 ± 0.09	9	9	9	9	9	9	9	9
	65–69	8.5 ± 0.09	9	9	9	9	9	9	9	9
	70–74	8.4 ± 0.08	9	9	9	9	9	9	9	9
	75–79	8.2 ± 0.09	9	9	9	9	9	9	9	9
	80 +	7.9 ± 0.16	9	9	9	9	9	9	9	9
Tni93 spatial recall (*n* = 2,987)	55–59	7.1 ± 1.7	−	−	6	7	−	−	−	−
	60–64	7.0 ± 1.7	−	−	6	7	−	−	−	−
	65–69	7.1 ± 1.8	−	−	6	7	−	−	−	−
	70–74	7.1 ± 1.7	−	−	6	7	−	−	−	−
	75–79	6.7 ± 1.8	−	−	6	7	−	−	−	−
	80 +	6.9 ± 1.9	−	−	6	7	−	−	−	−
Picture naming test (*n* = 2,993)	55–59	81.5 ± 6.6	74	76	77	78	−	90	−	−
	60–64	81.3 ± 6.6	75	78	84	87	−	90	−	−
	65–69	82.2 ± 6.7	75	78	84	87	−	90	−	−
	70–74	81.5 ± 6.7	73	76	77	78	−	90	−	−
	75–79	81.6 ± 7.0	73	75	76	78	−	90	−	−
	80 +	82.4 ± 7.2	75	78	84	87	−	90	−	−
Stick design test (*n* = 2,294)	55–59	22.2 ± 4.3	9	14	20	20	23	24	−	−
	60–64	22.2 ± 4.4	9	14	20	20	23	24	−	−
	65–69	22.4 ± 4.4	9	14	20	20	23	24	−	−
	70–74	21.8 ± 4.4	9	13	20	18	23	24	−	−
	75–79	22.1 ± 4.4	9	14	20	18	23	24	−	−
	80 +	22.5 ± 4.4	9	14	21	20	23	24	−	−

“–” indicates the model (qreg and bsqreg) did not converge for the respective quantile.

**TABLE 5:** Education-wise normative values of ICMR-NCTB test scores obtained from the appropriate quantile regression model among high-risk older-adults in rural-India.

ICMR-NCTB tests	Education	Mean NCTB test score ± standard deviation	Percentiles							
			5th	10th	15th	25th	50th	75th	90th	95th
Trail making test part A (*n* = 2,667)	Low or not literate	78.7 ± 14.6	159	126	116	104	85	64	60	50
	Literate	67.8 ± 17.1	132	116	107	94	75	62	48	40
Trail making test part B (*n* = 1,703)	Low or not literate	147.4 ± 29.5	259	237	224	205	154	117	89	80
	Literate	123.8 ± 33.0	193	162	146	126	100	87	70	64
CF vegetables (*n* = 2,998)	Low or not literate	10.4 ± 1.3	6	6	7	8	10	11	14	15
	Literate	11.1 ± 1.1	7	8	9	10	11	12	14	15
CF animals (*n* = 2,998)	Low or not literate	10.2 ± 1.8	6	7	8	9	10	11	13	14
	Literate	11.4 ± 1.4	8	9	10	10	12	12	13	14
CF food items (*n* = 2,998)	Low or not literate	9.3 ± 3.0	5	6	6	7	9	10	12	14
	Literate	9.1 ± 2.3	5	7	6	8	9	10	11	12
VLT total learning (*n* = 2,998)	Low or not literate	15.0 ± 1.7	7	9	10	13	15	19	21	23
	Literate	19.4 ± 1.8	13	14	15	16	19	22	25	26
VLT delayed recall (*n* = 2,998)	Low or not literate	4.6 ± 0.7	1	2	2	3	4	6	7	8
	Literate	6.1 ± 0.8	4	4	5	5	6	7	8	9
VLT delayed recognition (*n* = 2,999)	Low or not literate	19.1 ± 0.9	15	17	19	20	20	20	20	20
	Literate	19.3 ± 0.7	18	19	17	17	19	20	20	20
Tni93 total recall (*n* = 2,989)	Low or not literate	8.9 ± 0.08	9	9	9	9	9	9	9	9
	Literate	8.7 ± 0.09	9	9	9	9	9	9	9	9
Tni93 spatial recall (*n* = 2,987)	Low or not literate	7.0 ± 1.7	−	−	6	7	−	−	−	−
	Literate	7.3 ± 1.7	−	−	6	7	−	−	−	−
Picture naming test (*n* = 2,993)	Low or not literate	81.5 ± 6.7	75	78	84	87	−	90	−	−
	Literate	83.4 ± 6.9	74	76	77	81	−	92	−	−
Stick design test (*n* = 2,294)	Low or not literate	21.6 ± 4.4	8	12	15	18	20	24	−	−
	Literate	24.8 ± 3.3	16	20	21	22	23	27	−	−

“–” indicates the model (qreg and bsqreg) did not converge for the respective quantile.

**TABLE 6:** Education and age specific normative values of ICMR-NCTB test scores obtained from quantile regression model among high-risk older-adults in rural India.

ICMR-NCTB tests		Age (years)	Mean NCTB test score ± standard deviation	Percentiles							
				5th	10th	15th	25th	50th	75th	90th	95th
Trail making test part A	Low or not literate	55–64 (*n* = 1,041)	77.1 ± 12.8	151	124	113	103	79	65	60	52
		65–74 (*n* = 863)	78.1 ± 13.4	174	127	117	103	83	63	58	44
		75+ (*n* = 335)	78.8 ± 15.6	151	130	111	99	75	67	56	46
	Literate	55–64 (*n* = 200)	68.1 ± 22.1	123	113	106	100	84	67	63	53
		65–74 (*n* = 181)	76.9 ± 25.4	142	122	117	107	89	72	62	59
		75+ (*n* = 47)	70.7 ± 29.3	149	134	132	100	78	74	70	70
Trail making test part B	Low or not literate	55–64 (*n* = 680)	109.4.5 ± 15.8	253	224	211	201	149	114	83	76
		65–74 (*n* = 538)	147.3 ± 27.6	255	232	214	205	145	117	92	83
		75+ (*n* = 229)	150.7 ± 34.2	271	251	237	218	163	113	99	79
	Literate	55–64 (*n* = 126)	120.0 ± 44.9	200	147	123	102	93	74	65	63
		65–74 (*n* = 102)	132.3 ± 67.7	163	123	120	102	83	75	63	53
		75+ (*n* = 28)	122.4 ± 57.5	111	108	102	92	87	79	72	58
CF vegetables	Low or not literate	55–64 (*n* = 1,171)	10.4 ± 1.3	6	6	7	8	10	11	13	14
		65–74 (*n* = 993)	10.4 ± 1.3	6	7	7	8	10	11	14	15
		75+ (*n* = 397)	9.5 ± 1.2	5	6	6	7	9	11	14	15
	Literate	55–64 (*n* = 202)	11.1 ± 1.2	7	8	9	10	11	12	14	15
		65–74 (*n* = 186)	11.0 ± 1.0	7	8	8	9	11	12	−	−
		75+ (*n* = 49)	9.6 ± 0.5	−	8	8	10	10	10	13	−
CF animals	Low or not literate	55–64 (*n* = 1,171)	10.1 ± 1.7	6	7	7	9	10	11	13	14
		65–74 (*n* = 993)	9.8 ± 1.4	6	7	8	8	10	11	13	14
		75+ (*n* = 397)	9.9 ± 1.2	6	6	7	8	10	11	13	15
	Literate	55–64 (*n* = 202)	11.0 ± 1.8	9	10	10	11	12	13	14	15
		65–74 (*n* = 186)	11.6 ± 0.8	−	7	8	10	11	12	14	15
		75+ (*n* = 49)	10.9 ± 1.0	7	7	7	10	10	11	13	13
CF food items	Low or not literate	55–64 (*n* = 1,171)	9.5 ± 3.0	5	6	6	7	10	10	12	14
		65–74 (*n* = 993)	9.4 ± 3.0	5	6	6	7	9	10	12	13
		75+ (*n* = 397)	9.0 ± 2.4	5	6	6	7	8	10	11	13
	Literate	55–64 (*n* = 202)	9.2 ± 2.6	6	7	7	8	9	10	11	12
		65–74 (*n* = 186)	9.1 ± 2.4	6	7	7	8	9	10	10	12
		75+ (*n* = 49)	8.4 ± 2.1	5	6	8	8	9	9	10	11
VLT total learning	Low or not literate	55–64 (*n* = 1,173)	15.0 ± 1.7	8	9	10	12	15	19	21	23
		65–74 (*n* = 993)	15.5 ± 2.3	7	8	10	12	16	19	21	23
		75+ (*n* = 396)	15.2 ± 2.3	6	9	10	12	15	19	21	25
	Literate	55–64 (*n* = 202)	19.8 ± 2.1	12	15	15	16	19	22	24	27
		65–74 (*n* = 186)	19.1 ± 2.2	12	13	14	15	18	21	−	25
		75+ (*n* = 49)	19.8 ± 2.4	12	12	14	16	19	22	24	25
VLT delayed recall	Low or not literate	55–64 (*n* = 1,173)	4.9 ± 0.9	1	2	2	3	4	6	7	8
		65–74 (*n* = 993)	4.8 ± 0.9	1	2	2	3	4	6	7	8
		75+ (*n* = 395)	4.8 ± 0.9	0	1	2	3	4	6	7	8
	Literate	55–64 (*n* = 202)	6.2 ± 0.7	3	4	4	5	6	7	6	9
		65–74 (*n* = 186)	6.4 ± 1.4	4	4	4	5	6	7	8	−
		75+ (*n* = 49)	6.0 ± 1.8	3	4	5	5	5	7	8	8
VLT delayed recognition	Low or not literate	55–64 (*n* = 1,173)	−	15	18	19	−	−	−	−	−
		65–74 (*n* = 993)	−	16	17	19	−	−	−	−	−
		75+ (*n* = 396)	−	15	16	18	−	−	−	−	−
	Literate	55–64 (*n* = 202)	−	−	−	−	−	−	−	−	−
		65–74 (*n* = 186)	−	18	−	−	−	−	−	−	−
		75+ (*n* = 49)	−	19	19	19	−	−	−	−	−
Tni 93 total recall	Low or not literate	55–64 (*n* = 1,173)	−	−	−	−	−	−	−	−	−
		65–74 (*n* = 993)	−	−	−	−	−	−	−	−	−
		75+ (*n* = 396)	−	−	−	−	−	−	−	−	−
	Literate	55–64 (*n* = 202)	−	−	−	−	−	−	−	−	−
		65–74 (*n* = 186)	−	−	−	−	−	−	−	−	−
		75+ (*n* = 49)	−	−	−	−	−	−	−	−	−
Tni93 spatial recall	Low or not literate	55–64 (*n* = 1,166)	−	4	6	−	−	−	−	−	−
		65–74 (*n* = 988)	−	5	5	6	−	−	−	−	−
		75+ (*n* = 396)	−	4	5	5	−	8	9	9	−
	Literate	55–64 (*n* = 202)	−	−	8	−	−	−	−	−	−
		65–74 (*n* = 186)	−	−	−	−	−	−	−	−	−
		75+ (*n* = 49)	−	6	−	−	−	9	−	9	−
Picture naming test	Low or not literate	55–64 (*n* = 1,170)	87.7 ± 3.0	75	84	87	87	−	90	−	−
		65–74 (*n* = 990)	87.4 ± 3.0	72	73	77	82	−	88	−	−
		75+ (*n* = 397)	86.0 ± 3.6	69	75	78	86	90	90	−	−
	Literate	55–64 (*n* = 201)	−	84	85	−	−	−	−	−	−
		65–74 (*n* = 186)	−	87	−	88	−	−	−	−	−
		75+ (*n* = 49)	−	84	−	−	−	−	−	−	−
Stick design test	Low or not literate	55–64 (*n* = 893)	22.1 ± 4.0	8	9	16	18	20	25	27	−
		65–74 (*n* = 745)	21.2 ± 4.9	8	14	16	19	20	−	−	−
		75+ (*n* = 265)	21.2 ± 5.9	7	16	17	20	20	23	−	−
	Literate	55–64 (*n* = 185)	21.8 ± 4.4	16	20	22	23	26	30	−	−
		65–74 (*n* = 166)	22.1 ± 4.4	18	20	21	−	−	29	−	−
		75+ (*n* = 40)	22.5 ± 4.4	15	12	21	22	24	−	−	−

“–” indicates the model (qreg and bsqreg) did not converge for the respective quantile. The table consists of age and education stratified, gender and site adjusted values.

### 3.3 Choice of cut-offs

In neuropsychological assessment, normative cutoffs are intended for screening and early detection, supporting clinical interpretation rather than establishing diagnoses. While the 5th percentile may provide a clearer distinction between cognitively impaired and cognitively normal individuals, it may be overly conservative for screening. In a normal distribution, the 15th percentile corresponds to approximately −1.0 SD, a range typically characterized as *low* but not *clearly impaired*. Consequently, the 15th percentile is recommended as an appropriate cut-off because it balances sensitivity and specificity. In contrast, the 25th percentile (approximately −0.7 SD) is often considered liberal, resulting in lower specificity and a higher rate of false positives. However, because MCI is a fluid state, this study recommended that any individual measure falling at or below the 25th percentile indicated an increased likelihood of cognitive impairment. Although percentiles provide a framework for identifying low performance, the study also employed an ROC-based cut-off that more directly distinguished normal cognition from MCI/dementia.

The language-specific normative values are presented in Supplementary Tables S2–S4. Tests such as TNI93 total recall, TNI93 spatial recall, PNT, and stick design tests exhibited ceiling effects, with several participants achieving the maximum possible score, resulting in a dense and skewed upper tail of the distribution. The centiles for these tests were not derived from the BCPE approach due to a ceiling effect, highlighting the importance of quantile regression, which provides the 15th and 25th centiles (Tables 4, 5), a crucial screening threshold with immense clinical utility. The normative values from the BCPE approach are thus not presented and are kept as future scope.

### 3.4 Cognitive impairment status and the associated factors

Data from 3,175 participants were used to investigate the cross-sectional classification ability of the ICMR-NCTB test scores against the CDR-SOB. Baseline characteristics were also assessed by cognitive status, highlighting that subjects with early cognitive impairment (70.1 ± 8.9) were not older than those with normal cognition (66.1 ± 7.7). There was a disparity in education, with more low-literate subjects [172 (6.3%)] than literate subjects [03 (0.7%)]. As per gender, 4% men and 6% women were in the early cognitive impairment group. It is important to note that the asymptotic significances in Supplementary Table S5 were statistical, and the differences were not clinically important.

Further, the performance of these tests was assessed using the CDR classification. In classifying individuals as having Normal cognition (scored 1) or cognitively impaired (scored 0), the AuROC, cutoff, and the sensitivity and specificity are presented in Table 7.

**TABLE 7:** The classification ability of the ICMR-NCTB test scores in classifying the high-risk older-adults rural individuals in India.

ICMR-NCTB tests	AuROC (95%CI)	Cutoff	Sensitivity	Specificity
TMT A *(*n* = 2,771)	0.67 (0.63, 0.72)	< 79	75	51**
TMT B *(*n* = 1,805) ^#^	0.61 (0.56, 0.67)	< 157	54	59
CF vegetables (*n* = 3,172)	0.70 (0.66, 0.74)	> = 9	75	56
CF animals (*n* = 3,171)	0.70 (0.66, 0.74)	> = 9	74	53***
CF food items (*n* = 3,173)	0.52 (0.48, 0.57)	−	−	−
VLT total (*n* = 3,172)	0.83 (0.80, 0.86)	> = 13	75	75
VLT delayed recall (*n* = 3,171)	0.81 (0.77, 0.84)	> = 4	77	69
VLT delayed recognition (*n* = 3,172)	0.69 (0.64, 0.73)	> = 17	83	51
Tni 93 total recall (*n* = 3,141)	0.71 (0.66, 0.75)	> = 8	87	51
Tni93 spatial recall (*n* = 3,141)	0.71 (0.66, 0.76)	> = 6	86	50
Picture naming test (*n* = 3,168)	0.74 (0.69, 0.78)	> = 83	68	70
Stick design total score (*n* = 2,405)	0.61 (0.55, 0.67)	> = 19	71	51
Attention and executive function	0.67(0.63, 0.72)	> = −0.33	68	58
Episodic memory	0.83 (0.80,0.87)	> = −0.30	75	79
Language	0.72(0.67,0.76)	> = −0.13	68	68
Visuospatial	0.72(0.67,0.77)	> = −0.81	87	53
Composite Z-Score****	0.84(0.80,0.87)	> = −1.16	71	77

*Reverse coded. ^#^ Excluding Rajasthan. The cut-off favor normal cognition. ** The best cut-off according to Youden’s Index is > = 75; Sensitivity: 86%. Specificity: 46%; a cut-off of > = 79 was chosen to maintain an acceptable specificity. *** The best cut-off according to Youden’s Index is > = 8; Sensitivity: 85%. Specificity: 42%; a cut-off of > = 9 was chosen to maintain an acceptable specificity. ****A composite z-score is obtained by averaging the z-scores for each test (TMT-A and TMT-B being reverse coded) The best cut-off according to Youden’s Index is > = −0.41; Sensitivity: 83% specificity: 67%. A cut-off of > = −1.16 was chosen to maintain an acceptable specificity.

The ROC analysis revealed that most tests demonstrated acceptable to good classification performance with AuROCs up to 0.84, indicating that 84% of the cognitively normal individuals will have a composite score higher than those with MCI. As anticipated, cognitively healthy individuals took less time on TMT-A and TMT-B and scored higher on category fluency than cognitively impaired individuals. Similarly, patients with cognitive impairment performed poorly on the PNT, VLT (total learning and delayed recall), and stick design tasks, scoring lower than healthy individuals, suggesting difficulties in language, memory, and visuospatial abilities. The cut-offs investigated were approximately at the 25th percentile for most test scores in Table 4, with acceptable sensitivity and specificity for MCI overall and across languages. These empirically derived clinical cut-offs corresponded roughly to approximately 0.7 standard deviations below the normative mean, supporting the use of a 25th percentile cut-off for screening in Indian clinical and research settings. Upon investigating performance across languages, it was noted that in Hindi-speaking Rajasthan and Himachal Pradesh, no individuals with cognitive impairment were identified. The performance of the ICMR-NCTB test for Kannada and Bengali is presented in Supplementary Tables S6, S7.

## 4 Discussion

This study presents normative data and cut-offs for the ICMR-NCTB tests among high-risk older adults in rural India. In settings where linguistic diversity, low literacy, and limited access to specialist services hinder the recognition of cognitive impairment, the availability of contextually valid norms is crucial for accurately interpreting cognitive performance and for early identification of the impairment (Gill et al., 2020; He et al., 2022; Naja et al., 2017). Using data from a large multi-site rural cohort (SMRUTHI-INDIA), this study confirms that cognitive test score distributions are markedly non-normal, with substantial skewness and kurtosis across the majority of ICMR-NCTB measures, consistent with findings in other low-education populations (Freedman and Manly, 2015; Mitrushina et al., 2005). Across the ICMR-NCTB tests, unadjusted exploratory findings revealed that education had mild to moderate associations; however, age demonstrated no linear relationship, and the correlation with gender was minimal. Given that more than 85% of participants were either not literate or low-literate, the findings emphasize that failing to account for literacy may increase the false-positive diagnoses of cognitive impairment and are supported by existing literature (Kahali et al., 2023; Mannar et al., 2024; O’Connell et al., 2022; Sosa et al., 2009). The mild correlation with gender supports reporting combined-gender norms for the ICMR-NCTB domains in similar rural setting (Sosa et al., 2009). By applying quantile regression and an inter-percentile range–based model selection strategy, the study provided statistically robust centiles, particularly at lower percentiles, which were most relevant for clinical screening (O’Connell et al., 2022). This approach avoided the misclassification that could arise when mean-based norms were applied to skewed data and aligns with contemporary recommendations for normative modeling in aging cohorts (delCacho-Tena et al., 2024; O’Connell et al., 2022; Sachs et al., 2020). Centiles were also derived from the BCPE method, which needed less computation time and produced results consistent with reported cutoff values; however, generating norms for tests with ceiling effects remains challenging (McHorney and Tarlov, 1995; Terwee et al., 2007).

Previous community-based studies in India reported norms for diverse cognitive domains in 1,440 healthy individuals aged 45 and above, including illiterate, literate, and low-literate individuals, utilizing the COGNITO battery. Percentile scores for the cognitive tests declined among individuals aged 75 or older, and cognitive abilities were higher among literate individuals, followed by low- and non-literate individuals (Kahali et al., 2023). The present study findings were consistent with those of the SANSCOG study (Kahali et al., 2023), indicating age- and literacy-related changes in cognition (Menon et al., 2020; Sosa et al., 2009). The present study also revealed varied cut-offs across languages, consistent with previous studies (Verma et al., 2021) reporting the highest cut-offs for TMT A, TMT B, and CF Animal for Kannada speakers, followed by Bengali and Hindi. The present study proposes that individuals at or below the 25th percentile are more likely to have cognitive impairment. The 25th-percentile cut-offs for Kannada, Bengali, and Hindi align well with those reported for TMT A in previous studies (Verma et al., 2021). Category fluency norms in ICMR-NCTB (∼10 items) are lower than the global norms on NIH Toolbox (often more than 15 items) presented among English-speaking healthy adults and children (Casaletto et al., 2015), however, are close to another study presenting norms on The Uniform Data Set Version 3 Neuropsychological Battery (UDSNB 3.0) among community-residing English-speaking individuals (Wang et al., 2021) aged 70 years and more, as well as a rural community-based Indian study on an adapted CERAD tool among participants with comparable demographic characteristics (Ganguli et al., 1996). A similar pattern was noted for TMT A, TMT B, and VLT Total, reinforcing the importance of community-based, population-specific normative data. Performance on VLT Delayed Recall and Delayed Recognition remained stable across age groups, in contrast to Western studies reporting age-related decline after 70 years of age (Stricker et al., 2021), although it aligns with Indian community-based studies using adapted verbal learning tests, which report weaker age effects and ceiling effects in low-to-moderate education groups. Across most ICMR-NCTB tests, literates performed better than low-literate participants, with the largest differences observed in TMT-A, TMT-B, VLT learning, delayed recall, and stick design. In contrast, VLT delayed recognition, and TNI-93 total recall showed minimal literacy-related differences, limiting their clinical utility for detecting impairment. These findings support education-adjusted, percentile-based interpretation in community settings. A study (Menon et al., 2020) reported an optimal composite z-score cut-off of −0.54 (corresponding to the 30th percentile) with a sensitivity of 81.1% and a specificity of 88.8% for diagnosing all-cause MCI. However, in the present study, the optimal composite z-score cut-off was −1.16 (corresponding to the 12th percentile), with an area under the ROC curve (AUC) of 84% (95% CI: 80–87) and balanced sensitivity of 71.4% and specificity of 77.4%.

In normative cognitive research, a few tests exhibit ceiling effects, resulting in a dense and skewed upper tail of the distribution (Ganguli et al., 1996). While ceiling effects may not compromise initial screening, they can limit accurate classification of performance levels, thereby undermining the screening utility of such measures (Fogel, 1991; Storey and Kinsella, 2007). However, several measures, including delayed recognition, TNI-93 recall, picture naming and stick design, displayed ceiling effects due to restricted score ranges and limited percentile dispersion. Such ceiling effects, also reported in other international batteries adapted to the Indian context (Ganguli et al., 1996), reduce sensitivity to subtle age-related decline, particularly in highly educated or cognitively sound individuals. Quantile regression models effects across the entire score distribution, rather than on average, which is crucial when ceiling effects flatten the upper end of the distribution. While BCPE is designed to handle skewness, it is not robust to artificial truncation of the data at the maximum value (McHorney and Tarlov, 1995; Terwee et al., 2007). Extrapolation using quantile regression is computationally intensive yet less stable compared to the BCPE approach, especially for large datasets or a large number of predictors. Bootstrapping further increases computation time. Occasional non-convergence of the quantile regression model may be due to small sample sizes, especially for the education-age, language-age, and language-education-specific findings. Non-convergence was observed for variables with low variability, ceiling effects, or a discrete nature. The choice of model is left to the reader’s discretion and appropriateness.

The study features a large sample size, a multi-site rural setting, rigorous quality-control checks, and the use of advanced modeling techniques with normative data. The cohort consisted of high-risk older adults classified using CAIDE scores, which may limit generalizability to low-risk individuals (with low CAIDE scores or younger in age) and to those in urban India. Future studies reporting norms using follow-up data from the SMRUTHI cohort will be crucial for determining how the baseline centile spot predicts incident dementia cases and cognitive decline, and for validating it against clinical diagnoses (Van der Elst et al., 2013). Modifications to the BCPE framework and alternative approaches will be taken up as future scope.

## 5 Conclusion

The study provides age-, education-, gender-, and site-adjusted findings, along with age- and education-stratified, gender- and site-adjusted normative data for cognitive tests in a large community-based sample of high-risk older adults residing in rural India. Language-age- and language-education-specific findings are also presented. The full-sample centiles and cut-offs generated in this study provide a practical tool for distinguishing poor performance attributable to cognitive decline rather than to limited education, gender, or age. The normative data show a steady pattern across older age, with stable, domain-specific age-related changes rather than unexpected decline, and literates performing better than their low-literate counterparts. Language-age-wise percentile norms aid screening precision by distinguishing normal age-related variability from clinically meaningful impairment, especially in culturally diverse populations, where a norm developed in another population may be inappropriate. However, ceiling effects and occasional non-convergence of quantile regression models at extreme percentiles highlight limitations in detecting subtle cognitive change over time for some measures, and upper-tail performance should be interpreted with caution. Overall, the ICMR-NCTB is a robust tool for clinical assessment and community-based research, while underscoring the need for complementary measures when high sensitivity to early cognitive decline is required.

This study establishes a milestone for research on cognitive impairment among high-risk older adults in rural India with the well-defined normative values in consideration of principal determinants of cognition and the linguistic diversity in India. The normative values will allow policymakers to set thresholds for screening of early cognitive decline, ensuring fair and equitable identification among high-risk rural-residing older adults. Understanding the substantial educational and cultural heterogeneity in India, the implementation of regionally relevant normative standards will facilitate early identification, referral, intervention and likely prevention of advancement to greater stages of dementia even in low-resource settings.

## Data Availability

The raw data supporting the conclusions of this article will be made available by the authors, without undue reservation.
