# Supplementary material for: Creating cognitive normative data for rural Indian population
Source: Front Aging Neurosci. 2026 Jul 24;18:1838843. doi: 10.3389/fnagi.2026.1838843 (PMC13447306; doi:10.3389/fnagi.2026.1838843)
Supplement: Supplementary file 1 [file Data_Sheet_1.docx]

**Supplementary File**

| **S. No.** | **Content** | **Page No.** |
| --- | --- | --- |
| **1** | Gender and education-based distribution of participant characteristics among older adults in rural India | **1** |
| **2** | Normative values of ICMR-NCTB test scores obtained utilising quantile regression method among Hindi speaking high-risk elderly in rural India | **2 - 4** |
| **3** | Age-specific and education-specific normative values of ICMR-NCTB test scores obtained utilising quantile regression method among Kannada speaking high-risk elderly in rural India | **5 - 7** |
| **4** | Age-specific and education-specific normative values of ICMR-NCTB test scores obtained utilising quantile regression method among Bengali Speaking high-risk elderly in rural India | **8 - 9** |
| **5** | Participant characteristics across their cognitive impairment status based on clinical dementia rating (CDR-SOB) in rural India | **10** |
| **6** | The discriminative ability of the ICMR-NCTB test scores in classifying the high-risk elderly rural Kannada-speaking individuals in India | **11** |
| **7** | The discriminative ability of the ICMR-NCTB test scores in classifying the high-risk elderly rural Bengali speaking individuals in India | **12** |

**Table S1.** Gender and education-based distribution of participant characteristics among older adults in rural India

| **Characteristics** | | **All Subjects**  **(n= 3000)** | **Men(n=1114)** | **Women**  **(n=1886)** | **P** | **Illiterate and low literate (n=2563)** | **Literate (n=437)** | **P** |
| --- | --- | --- | --- | --- | --- | --- | --- | --- |
| Age (in Years) *Mean ± SD* | | 66.1 ± 7.8 | 66.8 ± 7.7 | 65.6 ± 7.7 | <0.001 | 66.1 ± 7.8 | 65.5 ± 7.3 | 0.015 |
| Age Group n (%) | 55-59 (n=677) | 677 (22.57) | 213 (19.12) | 464 (24.60) | 0.001 | 571 (22.28) | 106 (24.26) | 0.137 |
|  | 60-64 (n=698) | 698 (23.27) | 250 (22.44) | 448 (23.75) |  | 602 (23.49) | 96 (21.97) |  |
|  | 65-69 (n=686) | 686 (22.87) | 266 (23.88) | 420 (22.27) |  | 571 (22.28) | 115 (26.32) |  |
|  | 70-74(n=493) | 493 (16.43) | 187 (16.79) | 306 (16.22) |  | 422 (16.47) | 71 (16.25) |  |
|  | 75-79(n=263) | 263 (8.77) | 119 (10.68) | 144 (7.64) |  | 233 (9.09) | 30 (6.86) |  |
|  | 80 + (n=183) | 183 (6.10) | 79 (7.09) | 104 (5.51) |  | 164 (6.40) | 19 (4.35) |  |
| CAIDE *Mean ± SD* | | 8.2 ± 1.3 | 8.5 ± 1.3 | 8 ±1.2 | <0.001 | 8.3 ± 1.2 | 7.5 ± 1.2 | <0.001 |
| Diabetes | Yes | 486 (16.2) | 198 (17.77) | 288 (15.27) | 0.072 | 378 (14.75) | 108 (24.71) | <0.001 |
|  | No | 2514 (83.8) | 916 (82.23) | 1598 (84.73) |  | 2185 (85.25) | 329 (75.29) |  |
| CDR SOB  n (%) | Normal | 3000 (94.49) | 1114 (95.54) | 1886 (93.88) | 0.113 | 2563 (93.71) | 437 (99.32) | <0.001 |
|  | MCI | 161 (5.07) | 49 (4.2) | 112 (5.57) |  | 158 (5.78) | 3 (0.68) |  |
|  | Dementia | 14 (0.44) | 03 (0.26) | 11 (0.55) |  | 14 (0.51) | 0 (0.00) |  |

**Table S2.** Normative values of ICMR-NCTB test scores obtained utilising quantile regression method among Hindi speaking high-risk elderly in rural India (n=1498)

| **ICMR-NCTB**  **tests** | **Age (Yrs)/ education stratum N (%)** | **Percentile** | | | | | | | |
| --- | --- | --- | --- | --- | --- | --- | --- | --- | --- |
|  |  | **5^th^** | **10^th^** | **15^th^** | **25^th^** | **50^th^** | **75^th^** | **90^th^** | **95^th^** |
| **Trail Making Test Part A** (n=1406) | 55-64  (n=653) | 130 | 120 | 105 | 89 | 67 | 55 | 42 | 36 |
|  | 65-74  (n=545) | 130 | 104 | 92 | 81 | 66 | 53 | 41 | 34 |
|  | 75+  (n=208) | 140 | 113 | 95 | 80 | 63 | 51 | 41 | 36 |
|  | Low or not literate (n=1082) | 130 | 112 | 100 | 85 | 67 | 57 | 43 | 38 |
|  | Literate (n=324) | 128 | 112 | 105 | 92 | 70 | 42 | 33 | 29 |
| **Trail Making Test Part B** (n=498) | 55-64 (n=236) | 197 | 157 | 143 | 127 | 101 | 89 | 73 | 65 |
|  | 65-74 (n=173) | 187 | 153 | 134 | 123 | 97 | 87 | 76 | 67 |
|  | 75+ (n=89) | 182 | 134 | 123 | 111 | 99 | 89 | 81 | 78 |
|  | Low or not literate (n=342) | 187 | 157 | 143 | 126 | 101 | 89 | 79 | 77 |
|  | Literate (n=156) | 163 | 132 | 111 | 101 | 87 | 74 | 56 | 53 |
| **CF Vegetables** (n=1497) | 55-64  (n=690) | 6 | 7 | 7 | 8 | 10 | 11 | 13 | 14 |
|  | 65-74  (n=582) | 6 | 7 | 7 | 8 | 9 | 11 | 12 | 13 |
|  | 75+  (n=225) | 5 | 6 | 7 | 8 | 9 | 10 | 12 | 13 |
|  | Low or not literate (n=1168) | 6 | 7 | 7 | 8 | 9 | 11 | 12 | 13 |
|  | Literate (n=329) | 6 | 7 | 7 | 9 | 10 | 12 | 13 | 14 |
| **CF Animals**  (n=1497) | 55-64  (n=690) | 6 | 6 | 7 | 8 | 9 | 11 | 12 | 13 |
|  | 65-74  (n=582) | 5 | 6 | 7 | 8 | 9 | 10 | 11 | 12 |
|  | 75+  (n=225) | 5 | 6 | 7 | 8 | 9 | 11 | 12 | 13 |
|  | Low or not literate (n=1168) | 5 | 6 | 7 | 8 | 9 | 10 | 12 | 12 |
|  | Literate (n=329) | 6 | 7 | 8 | 9 | 11 | 12 | 14 | 14 |
| **CF Food Items** (n=1497) | 55-64  (n=690) | 4 | 4 | 5 | 5 | 7 | 8 | 10 | 11 |
|  | 65-74  (n=582) | 4 | 4 | 5 | 5 | 6 | 8 | 10 | 11 |
|  | 75+  (n=225) | 4 | 4 | 5 | 5 | 7 | 8 | 9 | 9 |
|  | Low or not literate (n=1168) | 4 | 4 | 5 | 5 | 6 | 8 | 9 | 10 |
|  | Literate (n=329) | 5 | 5 | 5 | 6 | 8 | 9 | 10 | 11 |
| **VLT Total Learning (TL) (T1 + T2 + T3)** (n=1498) | 55-64  (n=691) | 10 | 12 | 13 | 15 | 18 | 20 | 22 | 24 |
|  | 65-74  (n=582) | 11 | 12 | 13 | 15 | 17 | 20 | 22 | 23 |
|  | 75+  (n=225) | 12 | 14 | 14 | 15 | 17 | 20 | 22 | 23 |
|  | Low or not literate (n=1169) | 11 | 12 | 13 | 15 | 17 | 19 | 22 | 23 |
|  | Literate (n=329) | 13 | 15 | 16 | 18 | 20 | 23 | 25 | 26 |
| **VLT Delayed Recall** (n=1498) | 55-64  (n=691) | 3 | 3 | 4 | 5 | 6 | 7 | 8 | 8 |
|  | 65-74  (n=582) | 3 | 4 | 4 | 5 | 6 | 7 | 7 | 8 |
|  | 75+  (n=225) | 3 | 4 | 5 | 5 | 6 | 7 | 7 | 8 |
|  | Low or not literate (n=1169) | 3 | 4 | 4 | 5 | 6 | 7 | 7 | 8 |
|  | Literate (n=329) | 4 | 5 | 5 | 6 | 7 | 8 | 9 | 9 |
| **VLT Delayed recognition** (n=1498) | 55-64  (n=691) | 15 | 16 | 17 | - | - | - | - | - |
|  | 65-74  (n=582) | 16 | 17 | - | - | - | - | - | - |
|  | 75+  (n=225) | 15 | 16 | 16 | - | - | - | - | - |
|  | Low or not literate (n=1169) | 15 | 16 | 17 | - | - | - | - | - |
|  | Literate (n=329) | 19 | - | - | - | - | - | - | - |
| **Tni 93 total recall** (n=1498) | 55-64  (n=691) | 7 | 8 | 8 | - | - | - | - | - |
|  | 65-74  (n=582) | 7 | 8 | - | - | - | - | - | - |
|  | 75+  (n=225) | 7 | 7 | 7 | - | - | - | - | - |
|  | Low or not literate (n=1169) | 7 | 8 | 8 | - | - | - | - | - |
|  | Literate (n=329) | 8 | 8 | - | - | - | - | - | - |
| **Tni93 spatial recall** (n=1498) | 55-64  (n=691) | 5 | 6 | 6 | 7 | 9 | - | - | - |
|  | 65-74  (n=582) | 5 | 6 | 6 | 7 | - | - | - | - |
|  | 75+  (n=225) | 5 | 5 | 5 | 6 | - | - | - | - |
|  | Low or not literate (n=1169) | 5 | 5 | 6 | 6 | - | - | - | - |
|  | Literate (n=329) | 6 | 7 | 7 | 8 | - | - | - | - |
| **Picture naming test** (n=1492) | 55-64  (n=688) | 69 | 75 | 76 | 83 | 89 | 90 | 90 | 90 |
|  | 65-74  (n=579) | 69 | 74 | 76 | 81 | 90 | - | - | - |
|  | 75+  (n=225) | 65 | 69 | 72 | 74 | 85 | 90 | 90 | 90 |
|  | Low or not literate (n=1164) | 69 | 72 | 75 | 81 | 88 | - | - | - |
|  | Literate (n=328) | 84 | 87 | 88 | 90 | - | - | - | - |
| **Stick Design Test** (n=992) | 55-64  (n=474) | 9 | 15 | 17 | 20 | 22 | 25 | 30 | 30 |
|  | 65-74  (n=381) | 9 | 14 | 16 | 19 | 22 | 25 | 30 | 30 |
|  | 75+  (n=137) | 14 | 16 | 20 | 20 | 23 | 26 | 30 | 30 |
|  | Low or not literate (n=705) | 8 | 14 | 17 | 19 | 21 | 25 | - | - |
|  | Literate (n=287) | 16 | 20 | 21 | 22 | 24 | 27 | 30 | 30 |
| “-” indicates the model (qreg and bsqreg) did not converge for the respective quantile; The table consists of Language-age-specific gender-adjusted, and Language-education-specific gender-adjusted values | | | | | | | | | |

**Table S3.** Age-specific and education-specific normative values of ICMR-NCTB test scores obtained utilising quantile regression method among Kannada speaking high-risk elderly in rural India (n=992)

| **ICMR-NCTB**  **tests** | **Age (Yrs)/ education stratum N (%)** |  | **Percentile** | | | | | | |
| --- | --- | --- | --- | --- | --- | --- | --- | --- | --- |
|  |  | **5^th^** | **10^th^** | **15^th^** | **25^th^** | **50^th^** | **75^th^** | **90^th^** | **95^th^** |
| **Trail Making Test Part A** (n=780) | 55-64  (n=372) | 210 | 179 | 168 | 134 | 84 | 62 | 50 | 43 |
|  | 65-74  (n=322) | 211 | 182 | 165 | 121 | 83 | 62 | 52 | 44 |
|  | 75+  (n=86) | 209 | 181 | 168 | 148 | 81 | 68 | 62 | 59 |
|  | Low or not literate(n=720) | 210 | 181 | 168 | 134 | 81 | 62 | 52 | 44 |
|  | Literate (n=60) | 180 | 167 | 156 | 131 | 100 | 71 | 58 | 56 |
| **Trail Making Test Part B** (n=724) | 55-64  (n=354) | 297 | 289 | 275 | 234 | 150 | 111 | 88 | 79 |
|  | 65-74  (n=290) | 302 | 276 | 256 | 209 | 146 | 118 | 92 | 88 |
|  | 75+  (n=80) | 312 | 293 | 288 | 256 | 159 | 114 | 103 | 99 |
|  | Low or not literate(n=668) | 297 | 288 | 266 | 221 | 146 | 113 | 92 | 80 |
|  | Literate(n=56) | 361 | 354 | 314 | 299 | 222 | 187 | 157 | 128 |
| **CF Vegetables** (n=991) | 55-64  (n=455) | 8 | 9 | 10 | 11 | 13 | 15 | 16 | 17 |
|  | 65-74  (n=409) | 8 | 9 | 10 | 11 | 12 | 14 | 16 | 16 |
|  | 75+  (n=127) | 8 | 8 | 8 | 10 | 12 | 14 | 15 | 15 |
|  | Low or not literate(n=927) | 8 | 9 | 10 | 11 | 12 | 14 | 16 | 16 |
|  | Literate(n=64) | 8 | 9 | 10 | 10 | 12 | 13 | 16 | 17 |
| **CF Animals**  (n=991) | 55-64  (n=455) | 7 | 8 | 9 | 10 | 12 | 14 | 16 | 17 |
|  | 65-74  (n=409) | 7 | 8 | 8 | 10 | 12 | 14 | 15 | 16 |
|  | 75+  (n=127) | 7 | 8 | 9 | 10 | 11 | 13 | 15 | 15 |
|  | Low or not literate(n=927) | 7 | 8 | 9 | 10 | 12 | 14 | 15 | 16 |
|  | Literate(n=64) | 9 | 9 | 10 | 11 | 13 | 14 | 15 | 16 |
| **CF Food Items** (n=991) | 55-64  (n=455) | 9 | 10 | 11 | 12 | 13 | 15 | 16 | 18 |
|  | 65-74  (n=409) | 9 | 10 | 10 | 11 | 13 | 15 | 16 | 17 |
|  | 75+  (n=127) | 9 | 10 | 10 | 11 | 12 | 14 | 16 | 17 |
|  | Low or not literate(n=927) | 9 | 10 | 10 | 11 | 13 | 15 | 16 | 17 |
|  | Literate(n=64) | 11 | 11 | 12 | 13 | 14 | 15 | 19 | 21 |
| **VLT Total Learning (TL) (T1 + T2 + T3)** (n=991) | 55-64  (n=456) | 7 | 9 | 10 | 11 | 13 | 16 | 18 | 20 |
|  | 65-74  (n=409) | 7 | 8 | 9 | 10 | 13 | 15 | 17 | 19 |
|  | 75+  (n=126) | 6 | 8 | 9 | 10 | 13 | 14 | 17 | 17 |
|  | Low or not literate(n=927) | 7 | 8 | 9 | 10 | 13 | 15 | 18 | 19 |
|  | Literate(n=64) | - | 10 | 12 | 14 | 15 | 17 | 20 | 21 |
| **VLT Delayed Recall** (n=990) | 55-64  (n=456) | 1 | 2 | 2 | 3 | 4 | 5 | 6 | 7 |
|  | 65-74  (n=409) | 1 | 2 | 2 | 3 | 4 | 5 | 6 | 7 |
|  | 75+  (n=126) | 0 | 1 | 2 | 3 | 4 | 4 | 5 | 5 |
|  | Low or not literate(n=926) | 1 | 2 | 2 | 3 | 4 | 5 | 6 | 7 |
|  | Literate(n=64) | 1 | 2 | 2 | 3 | 4 | 6 | 6 | 7 |
| **VLT Delayed recognition** (n=991) | 55-64  (n=456) | 10 | 13 | 14 | 16 | 18 | 20 | 20 | 20 |
|  | 65-74  (n=409) | 10 | 11 | 14 | 15 | 18 | 20 | 20 | 20 |
|  | 75+  (n=126) | 10 | 10 | 12 | 15 | 17 | 19 | 20 | 20 |
|  | Low or not literate(n=927) | 10 | 12 | 14 | 15 | 18 | 20 | 20 | 20 |
|  | Literate(n=64) | 14 | 16 | 16 | 18 | 19 | 20 | 20 | - |
| **Tni 93 total recall** (n=991) | 55-64  (n=456) | - | - | - | - | - | - | - | - |
|  | 65-74  (n=408) | - | - | - | - | - | - | - | - |
|  | 75+  (n=127) | 9 | - | - | - | - | - | - | - |
|  | Low or not literate(n=927) | - | - | - | - | - | - | - | - |
|  | Literate(n=64) | - | - | - | - | - | - | - | - |
| **Tni93 spatial recall** (n=990) | 55-64  (n=456) | 4 | 6 | 6 | 7 | 9 | 9 | 9 | 9 |
|  | 65-74  (n=407) | 4 | 6 | 6 | 7 | 9 | 9 | 9 | 9 |
|  | 75+  (n=127) | 2 | 5 | 5 | 6 | 8 | 9 | 9 | - |
|  | Low or not literate(n=926) | 4 | 5 | 6 | 7 | 9 | 9 | 9 | 9 |
|  | Literate(n=64) | 4 | 7 | - | - | - | - | - | - |
| **Picture naming test** (n=992) | 55-64  (n=456) | 65 | 69 | 72 | 76 | 81 | 86 | 90 | 90 |
|  | 65-74  (n=409) | 61 | 65 | 69 | 72 | 78 | 85 | 89 | 90 |
|  | 75+  (n=127) | 64 | 66 | 68 | 72 | 78 | 83 | 87 | 88 |
|  | Low or not literate(n=928) | 63 | 67 | 70 | 74 | 79 | 84 | 89 | 90 |
|  | Literate(n=64) | 82 | 84 | 85 | 87 | 88 | 90 | 90 | - |
| **Stick Design Test** (n=929) | 55-64  (n=429) | 5 | 7 | 9 | 12 | 19 | 25 | 30 | 30 |
|  | 65-74  (n=387) | 5 | 7 | 8 | 11 | 17 | 22 | 27 | 30 |
|  | 75+  (n=113) | 4 | 5 | 6 | 10 | 15 | 23 | 27 | 30 |
|  | Low or not literate(n=866) | 5 | 6 | 8 | 11 | 18 | 23 | 29 | 30 |
|  | Literate(n=63) | 12 | 16 | 18 | 20 | 25 | 30 | 30 | 30 |
| “-” indicates the model (qreg and bsqreg) did not converge for the respective quantile; The table consists of Language-age-specific gender-adjusted, and Language-education-specific gender-adjusted values | | | | | | | | | |

**Table S4.** Age-specific and education-specific normative values of ICMR-NCTB test scores obtained utilising quantile regression method among Bengali Speaking high-risk elderly in rural India (n=510)

| **ICMR-NCTB**  **tests** | **Age (Yrs)/ education stratum N (%)** |  | **Percentile** | | | | | | |
| --- | --- | --- | --- | --- | --- | --- | --- | --- | --- |
|  |  | **5^th^** | **10^th^** | **15^th^** | **25^th^** | **50^th^** | **75^th^** | **90^th^** | **95^th^** |
| **Trail Making Test Part A** (n=481) | 55-64(n=216) | 149 | 123 | 117 | 105 | 92 | 79 | 70 | 66 |
|  | 65-74(n=177) | 174 | 143 | 117 | 109 | 99 | 87 | 76 | 69 |
|  | 75+(n=88) | 171 | 149 | 117 | 107 | 97 | 87 | 72 | 71 |
|  | Low or not literate(n=437) | 153 | 127 | 117 | 109 | 97 | 80 | 73 | 68 |
|  | Literate (n=44) | 149 | 117 | 101 | 99 | 93 | 81 | 79 | 77 |
| **Trail Making Test Part B** (n=481) | 55-64(n=216) | 240 | 217 | 210 | 200 | 180 | 127 | 99 | 97 |
|  | 65-74(n=177) | 249 | 241 | 219 | 212 | 175 | 125 | 107 | 101 |
|  | 75+(n=88) | 266 | 261 | 242 | 218 | 182 | 127 | 109 | 101 |
|  | Low or not literate(n=437) | 248 | 229 | 216 | 202 | 179 | 127 | 101 | 99 |
|  | Literate (n=44) | 253 | 237 | 211 | 180 | 131 | 114 | 95 | 93 |
| **CF Vegetables** (n=510) | 55-64(n=228) | 6 | 6 | 7 | 8 | 10 | 11 | 13 | 14 |
|  | 65-74(n=188) | 5 | 6 | 7 | 8 | 10 | 11 | 12 | 15 |
|  | 75+ (n=94) | 6 | 6 | 7 | 8 | 10 | 12 | 15 | 16 |
|  | Low or not literate(n=466) | 5 | 6 | 7 | 8 | 9 | 11 | 13 | 14 |
|  | Literate  (n=44) | 6 | 8 | 8 | 10 | 10 | 12 | 15 | 16 |
| **CF Animals**  (n=510) | 55-64(n=228) | 5 | 6 | 6 | 8 | 10 | 10 | 12 | 14 |
|  | 65-74(n=188) | 5 | 6 | 7 | 8 | 9 | 11 | 12 | 14 |
|  | 75+ (n=94) | 5 | 7 | 7 | 8 | 10 | 12 | 14 | 15 |
|  | Low or not literate(n=466) | 5 | 6 | 6 | 8 | 10 | 11 | 12 | 14 |
|  | Literate  (n=44) | 6 | 8 | 8 | 10 | 10 | 13 | 15 | 15 |
| **CF Food Items** (n=510) | 55-64(n=228) | 5 | 6 | 6 | 7 | 10 | 10 | 12 | 14 |
|  | 65-74(n=188) | 5 | 5 | 6 | 7 | 9 | 10 | 12 | 13 |
|  | 75+ (n=94) | 5 | 6 | 6 | 7 | 10 | 10 | 12 | 14 |
|  | Low or not literate(n=466) | 5 | 5 | 6 | 7 | 9 | 10 | 12 | 13 |
|  | Literate  (n=44) | 5 | 7 | 8 | 9 | 10 | 11 | 15 | 17 |
| **VLT Total Learning (TL) (T1 + T2 + T3)** (n=510) | 55-64(n=228) | 6 | 7 | 9 | 11 | 15 | 20 | 24 | 28 |
|  | 65-74(n=188) | 6 | 8 | 9 | 12 | 15 | 19 | 23 | 24 |
|  | 75+ (n=94) | 5 | 6 | 10 | 12 | 15 | 19 | 22 | 24 |
|  | Low or not literate(n=466) | 6 | 7 | 9 | 11 | 19 | 23 | 26 | 26 |
|  | Literate  (n=44) | 9 | 12 | 14 | 15 | 23 | 27 | 28 | 29 |
| **VLT Delayed Recall** (n=510) | 55-64(n=228) | 0 | 1 | 2 | 3 | 5 | 6 | 8 | 10 |
|  | 65-74(n=188) | 0 | 1 | 2 | 3 | 4 | 5 | 8 | 8 |
|  | 75+ (n=94) | 0 | 1 | 2 | 3 | 4 | 6 | 7 | 8 |
|  | Low or not literate(n=466) | 0 | 1 | 2 | 3 | 4 | 6 | 8 | 9 |
|  | Literate  (n=44) | 2 | 3 | 4 | 4 | 6 | 8 | 10 | 10 |
| **VLT Delayed recognition** (n=510) | 55-64(n=228) | 12 | - | - | - | - | - | - | - |
|  | 65-74(n=188) | 15 | - | - | - | - | - | - | - |
|  | 75+ (n=94) | 17 | 19 | 20 | - | - | - | - | - |
|  | Low or not literate(n=466) | 13 | 18 | - | - | - | - | - | - |
|  | Literate  (n=44) | - | - | - | - | - | - | - | - |
| **Tni 93 total recall** (n=500) | 55-64(n=221) | 3 | 4 | 4 | 5 | 7 | 9 | 9 | 9 |
|  | 65-74(n=186) | 3 | 4 | 4 | 5 | 6 | 8 | 9 | 9 |
|  | 75+ (n=93) | 4 | 5 | 5 | 6 | 7 | 9 | 9 | 9 |
|  | Low or not literate(n=456) | 3 | 4 | 4 | 5 | 6 | 8 | - | - |
|  | Literate(n=44) | 7 | 7 | - | - | - | - | - | - |
| **Tni93 spatial recall** (n=499) | 55-64(n=221) | 0 | 0 | 3 | 4 | 6 | 9 | 9 | 9 |
|  | 65-74(n=185) | 0 | 0 | 3 | 4 | 6 | 9 | 9 | 9 |
|  | 75+ (n=93) | 0 | 3 | 4 | 5 | 6 | 9 | 9 | 9 |
|  | Low or not literate(n=455) | 0 | 0 | 1 | 4 | 6 | 9 | 9 | 9 |
|  | Literate(n=44) | 4 | 4 | 5 | 6 | - | - | - | - |
| **Picture naming test** (n=509) | 55-64(n=227) | 75 | - | - | - | - | - | - | - |
|  | 65-74(n=188) | 72 | 75 | - | - | - | - | - | - |
|  | 75+ (n=94) | 78 | 84 | - | - | - | - | - | - |
|  | Low or not literate(n=465) | 60 | 75 | - | - | - | - | - | - |
|  | Literate | - | - | - | - | - | - | - | - |
| **Stick Design Test** (n=373) | 55-64 (n=175) | 13 | 18 | 22 | 27 | - | - | - | - |
|  | 65-74(n=143) | 7 | 14 | 17 | 24 | 30 | 30 | - | - |
|  | 75+ (n=55) | 14 | 20 | 24 | 27 | 30 | 30 | 30 | - |
|  | Low or not literate(n=332) | 9 | 15 | 19 | 25 | 30 | 30 | 30 | - |
|  | Literate(n=41) | 23 | 25 | - | - | - | - | - | - |
| “-” indicates the model (qreg and bsqreg) did not converge for the respective quantile; The table consists of Language-age-specific gender-adjusted, and Language-education-specific gender-adjusted values | | | | | | | | | |

The baseline characteristics were also assessed based on the participants' cognitive status presented in Table S5.

**Table S5** Participant characteristics across their cognitive impairment status based on clinical dementia rating (CDR-SOB) in rural India (n=3175)

| Characteristic | | Normal cognition (n=3000) | Early cognitive impairment (n=175) | P value |
| --- | --- | --- | --- | --- |
| Age (in years) *Mean ± SD* | | 66.05 ± 7.7 | 70.08 ± 8.9 | <0.001 |
| Age groups (in years) | 55-59 | 677 (96.71) | 23 (3.29) | <0.001 |
|  | 60-64 | 698 (96.54) | 25 (3.46) |  |
|  | 65-69 | 686 (94.88) | 37 (5.12) |  |
|  | 70-74 | 493 (93.90) | 32 (6.10) |  |
|  | 75-79 | 263 (88.85) | 33 (11.15) |  |
|  | 80 + | 183 (87.98) | 25 (12.02) |  |
| Gender | Males | 1114 (95.54) | 52 (4.46) | 0.048 |
|  | Females | 1886 (93.88) | 123 (6.12) |  |
| Education | Low or not literate | 2563 (93.71) | 172 (6.29) | <0.001 |
|  | Literate | 437 (99.32) | 03(0.68) |  |
| CAIDE score | Mean *±* SD | 8.2 ± 1.3 | 8.5 ± 1.3 | <0.001 |
|  | Range | 6,13 | 6,12 |  |

Upon investigating the performances for each language, it was noted that in Hindi-speaking Rajasthan and Himachal, there were none with cognitive impairment. The performance of ICMR-NCTB test for Kannada and Bengali are presented in Table S6 and S7.

Table S6. The discriminative ability of the ICMR-NCTB test scores in classifying the high-risk elderly rural Kannada-speaking individuals in India

| ICMR-NCTB tests | AuROC(95%CI) | Cutoff | Sensitivity | Specificity |
| --- | --- | --- | --- | --- |
| TMT A *(n=823) | 0.55(0.47,0.63) | >=103 | 51.16 | 60.00 |
| TMT B *(n=765) | 0.47(0.38,0.56) | -- | -- | -- |
| CF Vegetables (n=1072) | 0.74(0.68,0.78) | >=11 | 72.86 | 61.73 |
| CF Animals (n=1072) | 0.73(0.71,0.83) | >=11 | 69.63 | 61.73 |
| CF Food Items (n=1073) | 0.66(0.60,0.72) | >=13 | 60.04 | 58.54 |
| VLT Trial Total (n=1071) | 0.66(0.59,0.72) | >=12 | 65.79 | 56.25 |
| VLT Delayed Recall (n=1070) | 0.62(0.55, 0.68) | >=4 | 59.09 | 57.50 |
| VLT Delayed recognition (n=1071) | 0.62(0.56,0.68) | >=17 | 66.30 | 53.75 |
| Tni 93 total recall (n=1068) | 0.59(0.54,0.63) | -- | -- | -- |
| Tni93 spatial recall (n=1067) | 0.59(0.52,0.66) | >=9 | 54.34 | 53.25 |
| Picture naming test (n=1074) | 0.76(0.69,0.84) | >=80 | 70.41 | 77.55 |
| Stick Design total score (n=996) | 0.58(0.50,0.66) | >=17 | 57.37 | 53.73 |
| *Reverse coded, Normal (0); cognitively impaired (1) | | | | |

Table S7. The discriminative ability of the ICMR-NCTB test scores in classifying the high-risk elderly rural Bengali speaking individuals in India

| ICMR-NCTB tests | AuROC(95%CI) | Cutoff | Sensitivity | Specificity |
| --- | --- | --- | --- | --- |
| TMT A *(n=542) | 0.56(0.47,0.65) | >=98 | 59.02^#^ | 53.43 |
| TMT B *(n=542) | 0.58(0.50,0.66) | >=191 | 50.82 | 66.53 |
| CF Vegetables (n=603) | 0.74(0.68,0.80) | >=8 | 79.80 | 59.41 |
| CF Animals (n=602) | 0.78(0.72,0.83) | >=7 | 89.22 | 52.17 |
| CF Food Items (n=603) | 0.74(0.68,0.80) | >=7 | 84.90 | 51.61 |
| VLT Trial Total (n=603) | 0.83(0.78,0.88) | >=12 | 79.22 | 75.27 |
| VLT Delayed Recall (n=603) | 0.80(0.74, 0.85) | >=3 | 82.55 | 63.44 |
| VLT Delayed recognition (n=603) | 0.70(0.64,0.76) | >=16 | 95.29 | 47.31 |
| Tni 93 total recall (n=575) | 0.76(0.71,0.82) | >=7 | 61.60 | 74.67 |
| Tni93 spatial recall (n=576) | 0.71(0.65,0.78) | >=5 | 74.75 | 63.64 |
| Picture naming test (n=602) | 0.72(0.67,0.78) | >=84 | 88.61 | 51.61 |
| Stick Design total score (n=417) | 0.72(0.63,0.80) | >=27 | 72.92 | 63.64 |
| *Reverse coded, Normal (0); cognitively impaired (1)  ^#^ The best cutoff according to Youden’s Index is >=109; Sensitivity: 42.62%;  Specificity: 77.97% but a cutoff >=99 was chosen to maintain an acceptable sensitivity. | | | | |
